# Dual Transient Networks of Polymer and Micellar Chains: Structure and Viscoelastic Synergy

**DOI:** 10.3390/polym13234255

**Published:** 2021-12-04

**Authors:** Sébastien Roland, Guillaume Miquelard-Garnier, Andrey V. Shibaev, Anna L. Aleshina, Alexis Chennevière, Olga Matsarskaia, Cyrille Sollogoub, Olga E. Philippova, Ilias Iliopoulos

**Affiliations:** 1 Laboratoire PIMM, Arts et Metiers Institute of Technology, CNRS, Cnam, HESAM Universite, 75013 Paris, France; Sebastien.Roland@ensam.eu (S.R.); guillaume.miquelardgarnier@lecnam.net (G.M.-G.); cyrille.sollogoub@lecnam.net (C.S.); 2 Physics Department, Lomonosov Moscow State University, 119991 Moscow, Russia; shibaev@polly.phys.msu.ru (A.V.S.); aleshina@polly.phys.msu.ru (A.L.A.); 3 Laboratoire Léon Brillouin, CEA Saclay, 91191 Gif-sur-Yvette, France; alexis.chenneviere@cea.fr; 4 Institut Laue-Langevin, 38042 Grenoble, France; matsarskaia@ill.fr

**Keywords:** wormlike surfactant micelles, rheology, viscoelasticity, self-assembly, hydroxypropyl guar

## Abstract

Dual transient networks were prepared by mixing highly charged long wormlike micelles of surfactants with polysaccharide chains of hydroxypropyl guar above the entanglement concentration for each of the components. The wormlike micelles were composed of two oppositely charged surfactants potassium oleate and n-octyltrimethylammonium bromide with a large excess of anionic surfactant. The system is macroscopically homogeneous over a wide range of polymer and surfactant concentrations, which is attributed to a stabilizing effect of surfactants counterions that try to occupy as much volume as possible in order to gain in translational entropy. At the same time, by small-angle neutron scattering (SANS) combined with ultrasmall-angle neutron scattering (USANS), a microphase separation with the formation of polymer-rich and surfactant-rich domains was detected. Rheological studies in the linear viscoelastic regime revealed a synergistic 180-fold enhancement of viscosity and 65-fold increase of the longest relaxation time in comparison with the individual components. This effect was attributed to the local increase in concentration of both components trying to avoid contact with each other, which makes the micelles longer and increases the number of intermicellar and interpolymer entanglements. The enhanced rheological properties of this novel system based on industrially important polymer hold great potential for applications in personal care products, oil recovery and many other fields.

## 1. Introduction

Viscoelastic surfactants are able to self-assemble into very long wormlike micelles (WLMs) [1,2,3]. These micellar chains can entangle with each other forming a transient network that exhibits a viscoelastic behavior. Since WLMs are “living” objects that continuously break and reassemble, they easily change their structure to adapt to the variation of the external conditions. This makes their viscoelastic properties easily tunable by many triggers including temperature, shear, and different additives [4,5,6,7,8]. Responsive viscoelasticity of WLM solutions is widely used in a diverse range of applications from personal care products to oil recovery [1,2,3,9,10,11,12,13].

For some uses it is desirable to enhance the viscoelastic properties of WLMs while keeping their responsiveness. This can be done by mixing WLMs with polymeric chains [14,15,16,17,18,19,20,21,22,23,24,25]. Many combinations of polymers and WLMs have been studied up to now. Most often polymers binding to WLMs through electrostatic [16,19,20,25] or hydrophobic [14,15,17,18,22,23] interactions or both [21] were used. For instance, the addition of cationically modified cellulose to WLMs of anionic surfactants sodium dodecylbenzenesulfonate or sodium dodecylethoxysulfate was shown to induce a very pronounced increase of viscosity by few orders of magnitude resulting from electrostatic attractive interactions between the components [19]. However, oppositely charged polymer-surfactant mixture usually have a strong tendency for phase separation [16,19]. Alternative to electrostatic forces, one can exploit hydrophobic interactions between polymer and micellar chains using water-soluble polymers bearing few hydrophobic side [14,15,22,23] or end [17,18] groups. For instance, the addition of hydrophobically modified guar to WLMs of cationic surfactant erucyl bis(hydroxyethyl)methylammonium chloride was shown to increase the viscosity by few orders of magnitude [14]. This effect was attributed to cross-linking of the WLMs by polymer through intercalation of its hydrophobic side groups into the micellar core.

Multiple attempts to enhance the viscosity of WLMs by adding water-soluble polymers without oppositely charged or hydrophobic groups were ineffective [26,27,28,29]. They either did not produce any appreciable impact on viscosity [26,29] or even reduced it [26,27,29]. The latter effect was observed for such polymers as poly(propylene oxide) or poly(vinyl methyl ether), which adsorbed on the surface of WLMs thereby destroying them [26,27,29].

Recently it was found that even widely available water-soluble polymers that do not interact with micellar chains can enhance the viscoelasticity of WLMs, if they form their own network in the whole volume of the solution independently of the network of entangled WLMs [30,31]. In this system containing non-interacting components it is important to ensure the phase compatibility. This can be done by using strongly charged WLMs, since demixing is highly unfavorable for their counterions, which need to move over the entire system in order to gain translational entropy. It was demonstrated that addition of uncharged synthetic polymer-poly(vinyl alcohol) (PVA)-to the solution of highly charged mixed WLMs of potassium oleate and n-octyltrimethylammonium bromide C_8_TAB containing large excess of anionic surfactant can induce a 100-fold increase of viscosity [30,31].

A synergistic enhancement of viscosity upon mixing polymer and micellar chains gives the possibility to significantly reduce the amount of chemicals required to achieve a desired rheological profile, thereby lowering both cost and environmental impact. When considering the pertinence of such systems for oil recovery, it is important to check the applicability of this approach to polymers commonly used in this field. These polymers comprise polyacrylamide derivatives and various polysaccharides like xanthan, hydroxyethylcellulose, guar gum [32,33,34] and so on. Since the oil industry must meet the demand for environmental sustainability [35], the use of polymers from renewable sources (of which polysaccharides are a part) is preferable.

Among different polysaccharides applied in the petroleum industry, guar gum and its derivatives are of particular interest, since they account for possibly 90% of all gelled fracturing fluids in oil recovery [36]. Guar gum, produced from the seeds of *Cyamopsis tetragonalobus* plant native to India and Pakistan, is a nonionic polysaccharide with a backbone consisting of β-(1–4)-d-mannose residues to which α-(1–6)-d-galactopyranosyl units are attached as side groups [37]. This is a biocompatible, biodegradable, and non-toxic polymer [38]. To improve the hydration of guar gum at ambient temperatures, it can be chemically modified yielding various better water-soluble derivatives [39,40]. Among them, hydroxypropyl guar (HPG) is the most widely available [41] and, therefore, represents one of the most promising candidates for the preparation of the mixed systems with WLMs.

The aim of the present paper is to elaborate the dual transient networks in which HPG chains form their own network in the whole volume of the solution in addition to the network of entangled WLMs. The WLMs were composed of anionic surfactant potassium oleate mixed with a small amount of a cationic surfactant C_8_TAB so that the micelles were highly negatively charged. It was shown that despite the high molecular weight of the polymer the system does not phase separate in a wide range of concentrations of both polymer and surfactants. At the same time, the structural studies by small-angle neutron scattering (SANS) and ultrasmall-angle neutron scattering (USANS) revealed the microphase separation leading to locally concentrating the components in polymer-rich and surfactant-rich domains, which is responsible for the synergistic enhancement of the rheological properties. Such behavior makes these systems promising for various applications, for instance, in personal care products or for the creation of novel fracturing fluids for oil recovery.

## 2. Materials and Methods

### 2.1. Materials

**Figure 1:**
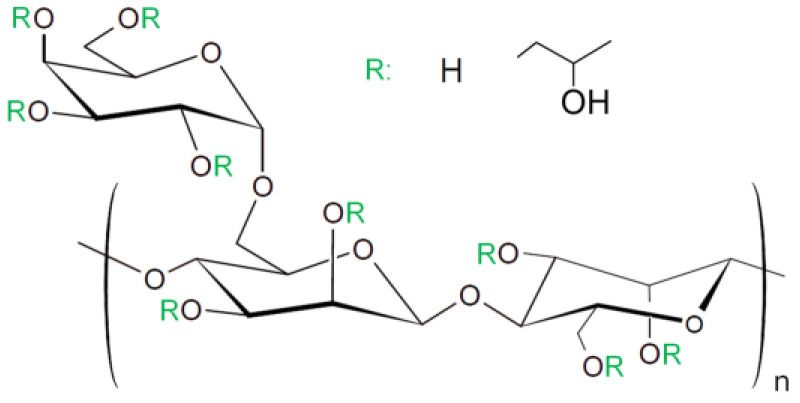
Chemical structure of HPG.

HPG (Jaguar^®^ HP-105) was kindly provided by Solvay and used without further purification. Its chemical structure is presented in Figure 1. The molar mass (Mw) of HPG was estimated as 1,600,000 g/mol from the value of intrinsic viscosity [η] in water ([η] = 12.7 dL/g) using Mark-Houwink-Sakurada relationship with K = 1.72 × 10^−4^ dL/g and a = 0.785 according to [41]. The relative proportion of galactose to mannose units was determined from ^1^H NMR spectra by integrating the peaks corresponding to galactose (5.02 ppm) and mannose (4.73 ppm) protons [37]. It was found that the sample contains on average 0.7 galactose side groups per one mannose unit of the backbone. The molar degree of substitution (the average number of moles of hydroxypropyl substituents per mole of saccharide units) was equal to 0.91 as estimated by ^1^H NMR from the peak integral of hydroxypropyl group methylene protons (1.15 ppm) divided by a sum of diether (–O–CH–O–) proton signals of mannose and galactose saccharide units (4.73–5.18 ppm). The incorporation of hydroxypropyl groups can occur at any of the hydroxyl groups on the chain, either on the backbone or on the side group [40]. It blocks some of the hydrogen bonding sites in macromolecules and thereby reduces the attractive interactions between polysaccharide chains, which improves their water solubility.

Potassium oleate from TCI (purity > 98%), C_8_TAB from ABCR (purity > 98%) and potassium hydroxide from Acros (purity > 98%) were used as received. The solutions were prepared using distilled deionized water. For SANS measurements D_2_O (99.96% isotopic purity) supplied by Sigma-Aldrich was used as a solvent.

### 2.2. Preparation of Samples

First, stock solutions of HPG (3 wt%), potassium oleate (15 wt%) and C_8_TAB (5 wt%) were prepared by dissolving the reagents in water. HPG and surfactants stock solutions were mixed by a magnetic stirrer for at least 24 and 12 h, respectively. Both solutions were perfectly transparent. Then, pH of all stock solutions was adjusted to 11.0 by adding 5 M KOH. High pH value is necessary to ensure the formation of cylindrical micelles in the final samples, which occurs only when a significant amount of oleate molecules are deprotonated [42] (pKa of oleic acid is equal to 9.85 [43]). The stock solutions were mixed in appropriate quantities to obtain the HPG/potassium oleate/C_8_TAB samples, 0.001 M KOH in water being used as a solvent. The [potassium oleate]/[C_8_TAB] ratio was always fixed at 2.5.

### 2.3. Phase Behavior

The partial phase diagram of HPG/potassium oleate/C_8_TAB/water system was constructed at 25 °C by visual inspection of the samples 5 days after preparation.

### 2.4. Small-Angle Neutron Scattering (SANS)

SANS experiments were performed on the PAXY facility at the Laboratoire Léon Brillouin (LLB) in Saclay and at the instrument D11 [44] of the Institut Laue-Langevin (ILL) in Grenoble, France. The samples in D_2_O were prepared at pD = 11.0 ± 0.2. Samples with low viscosity were examined in 2 mm quartz cells (Hellma, Germany). Viscous samples were studied in dismountable cells consisting of two round quartz windows separated by 1.45- or 2-mm teflon spacers.

At LLB, three different sample-to-detector distances of 1 m, 3 m and 5 m were used together with a neutron wavelength of 4 Å, 5 Å and 8.5 Å, respectively. The q range available was thus 0.006–0.6 Å^−1^. Standard corrections were applied for sample volume, neutron beam transmission, empty cell signal, and detector efficiency to the raw signal to obtain scattering spectra in absolute units [45].

At ILL, a q range of 7.63 × 10^−4^–0.65 Å^−1^ was covered by three sample-to-detector distances: 1.7 m, 16 m and 38 m. The wavelengths used were 4.6 Å at 1.7 m and 16 m and 13 Å at 38 m, with a full width-half maximum (FWHM) wavelength spread of 9%. Scattered neutrons were detected using a multitube ^3^He gas detector having a pixel size of 4 × 8 mm^2^. Calibration to absolute scale was performed using attenuated direct beam measurements. Raw data were saved in the nxs (NeXuS) format [46]. All data were corrected for transmission and background scattering.

The scattering length densities (SLD) of surfactant alkyl tails and D_2_O are equal to −0.3 × 10^−6^ Å^−2^ and 6.36 × 10^−6^ Å^−2^, respectively. The SLD of HPG can be estimated as 1.1 × 10^−6^ Å^−2^, but it may increase up to 4 × 10^−6^ Å^−2^ due to the substitution of the hydroxyl group protons by deuterium [47].

Fitting of the scattering curves was performed by the program SasView (http://www.sasview.org/; accessed on 7 April 2021). For fitting, only a part of the curve at q higher than the structure peak position q* (q > 0.06 Å^−1^) was used, and then the fit was reconstructed in the whole q-range. Two fitting parameters were used: radius and background. The length of the cylinder was set at a very large value (10,000 Å) much larger than the length scales in the accessible q-range.

### 2.5. Rheology

Rheological measurements were carried out on a stress-controlled rotational rheometer Anton Paar Physica MCR 301 (Graz, Austria) with cone-plate geometry (diameter of 50 mm, cone angle 1°) and a solvent trap to prevent water evaporation. Temperature was maintained at 20.00 + 0.05 °C by Peltier elements. The samples were equilibrated for 10–30 min in the measurement cell prior to investigation. The details of the measurements are described elsewhere [48,49,50].

In steady shear experiments, the dependences of viscosity on shear rate (flow curves) were measured in the range of shear rates from 0.001 to 200 s^−1^. In oscillatory shear experiments, the angular frequency dependences of the storage G′ (ω) and loss G″ (ω) moduli were measured in the linear viscoelastic regime, which was determined preliminarily by amplitude sweep tests.

### 2.6. NMR Spectroscopy

^1^H NMR measurements were carried out on a Bruker AV-600 spectrometer (Billerica, MA, USA) at 70 °C in D_2_O as a solvent at pD = 11.3 [51]. ^1^H chemical shifts were referenced to the HOD signal at 4.30 ppm.

## 3. Results and Discussion

### 3.1. Critical Concentrations of HPG and Surfactants Solutions

**Figure 2:**
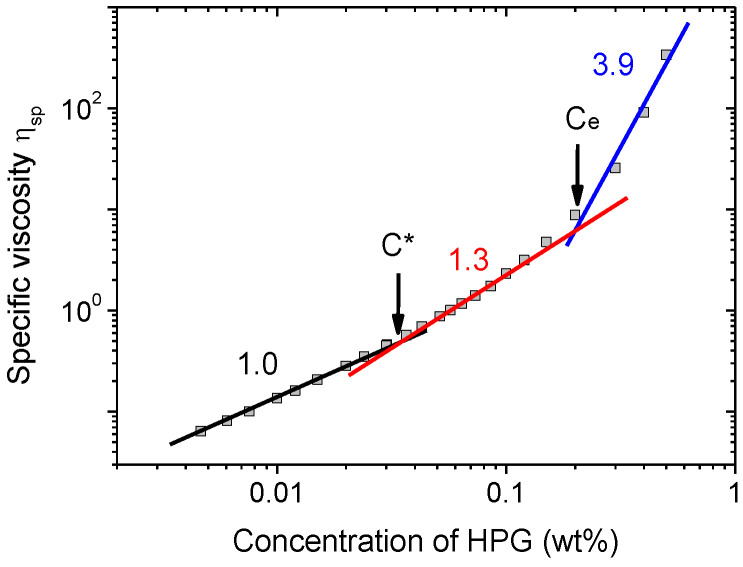
Dependence of the zero-shear specific viscosity on HPG concentration at 25 °C. C* denotes the overlap concentration, C_e_ is the entanglement concentration corresponding to the transition from the unentangled to entangled semi-dilute solution.

First, HPG solutions without WLMs were studied. Figure 2 shows the concentration dependence of the zero-shear specific viscosity η_sp_ of these solutions. Three regimes can be identified according to the values of exponents of power law dependence of η_sp_ on the polymer concentration C. At small HPG concentrations, a power law dependence η_sp_ ~ C^1^ is consistent with that expected for dilute solutions (η_sp_ ~ C^1^ [52]). The second (η_sp_ ~ C^1.3^) and third (η_sp_ ~ C^3.9^) regimes have the exponents matching the theoretical predictions (1.3 and 3.9 [52]) for unentangled and entangled semi-dilute solutions of uncharged polymer in good solvent, respectively. These exponents are close to those previously determined experimentally for HPG solutions in water: 1.25 [39] (second regime) and 3.12 [39], 4.3 [53], 4.5 [54] (third regime). Figure 2 allows determining critical concentrations of HPG solutions: the overlap concentration corresponding to the transition from dilute to semidilute regime C* = 0.03 wt.% and the entanglement concentration corresponding to the transition from unentangled to entangled semidilute regime C_e_ = 0.2 wt%.

The overlap concentration C*_WLM_ of the WLMs formed by potassium oleate/C_8_TAB surfactant mixture (at [potassium oleate]/[C_8_TAB] molar ratio equal to 2.5) is ca. 2 wt% (comprising 1.5 wt% potassium oleate and 0.5 wt% C_8_TAB) as was determined previously [30] from the sharp rise on the concentration dependence of zero-shear viscosity of the surfactant solutions. Above C*_WLM_ the solution contains long WLMs (their length goes up to a few micrometers [55]) entangled with each other.

Further experiments were performed at polymer and surfactants concentrations corresponding to a semidilute entangled regime to ensure the formation of a transient network of entanglements by each of the components of the dual HPG/surfactants network.

### 3.2. Phase Behavior of HPG/Surfactant System

**Figure 3:**
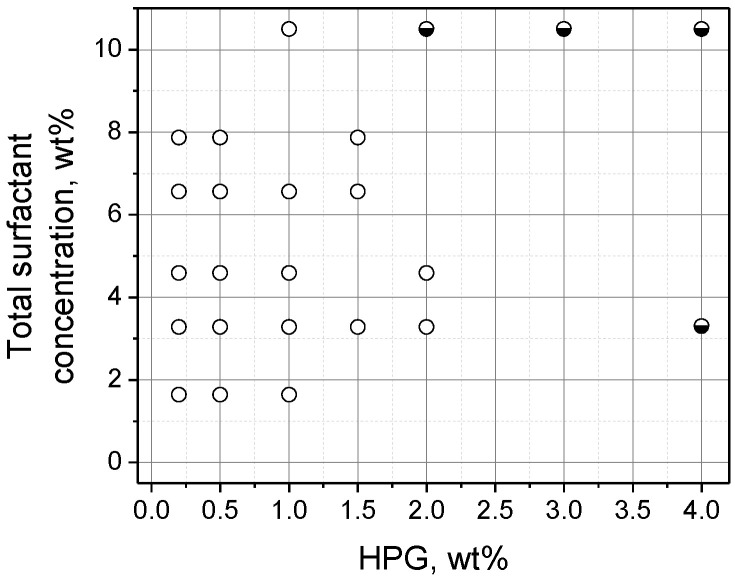
Partial phase diagram for HPG/potassium oleate/C_8_TAB/water system at 25 °C. The open symbols denote transparent homogeneous 1-phase systems, semi-filled symbols–2-phase systems. The [potassium oleate]/[C_8_TAB] ratio was fixed at 2.5.

The partial phase diagram of HPG/potassium oleate/C_8_TAB/water system is presented in Figure 3. It covers the range of HPG concentrations from 0.2 to 4 wt% (0.0027–0.054 monomol/L) corresponding to an entangled semi-dilute regime of pure polymer solution. The total concentration of both surfactants was varied from 1.6 to 10.5 wt% lying also mostly in the semi-dilute regime.

From the phase diagram (Figure 3) one can see that the solutions are homogeneous in a wide range of studied polymer and surfactant concentrations. Most probably, this is related to the presence of highly charged WLMs that try to avoid macrophase separation and occupy most of the volume of the system in order to gain in the translational entropy of their counterions.

**Figure 4:**
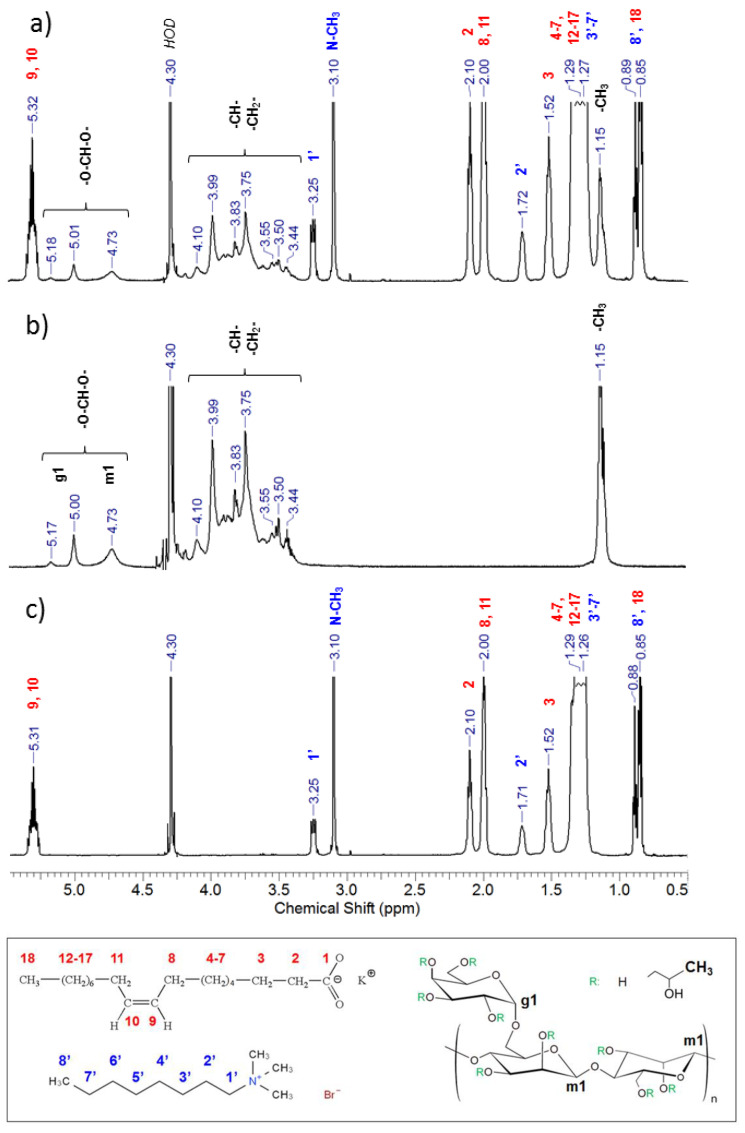
^1^H NMR spectra of (**a**) HPG/potassium oleate/C_8_TAB system and its components: (**b**) HPG and (**c**) potassium oleate/C_8_TAB mixture. Concentrations: 0.7 wt% HPG, 1.25 wt% potassium oleate, 0.395 wt% C_8_TAB (molar ratio [potassium oleate]/[C_8_TAB] = 2.5). Solvent: D_2_O. Temperature: 70 °C. Peak assignments were made according to the data reported in [40] and on www.chemicalbook.com (accessed on 1 April 2021) (SpectrumEN_143-18-0_1HNMR and SpectrumEN_2083-68-3_1HNMR).

Surprisingly, the 1-phase region in the present system is as wide as in the corresponding system containing PVA (Mw = 27,000 g/mol) [30] instead of HPG, although Mw of HPG is 60-fold greater. At the same time, it was established [56] that the compatibility of polymer blends decreases with increasing chain length of polymers, since it reduces the entropy of mixing. One could suggest that in the present system, the good compatibility may be related to some specific interactions occurring between HPG and WLMs. To check this suggestion the ^1^H NMR spectra of HPG/surfactant system were compared with those of its components taken separately (Figure 4). No differences in the chemical shifts of the signals (Figure 4) or their integrals (Table 1) were detected indicating that in the mixture HPG and the WLMs do not reside in close proximity to each other.

Most probably, a better compatibility of HPG with WLMs in aqueous medium as compared to PVA/WLMs/water system is related to a better water solubility of HPG. Indeed, HPG exhibits a lower Huggins coefficient in water (k_H_ = 0.32 [41]) than PVA (k_H_ = 0.51 [57]). Thus, in aqueous medium HPG has a weaker tendency to interchain interactions than PVA and therefore, it is less prone to the segregative phase separation. A large range of compatibility in HPG/surfactant system is very favorable for their potential applications.

### 3.3. Structure of HPG/Surfactant System

**Figure 5:**
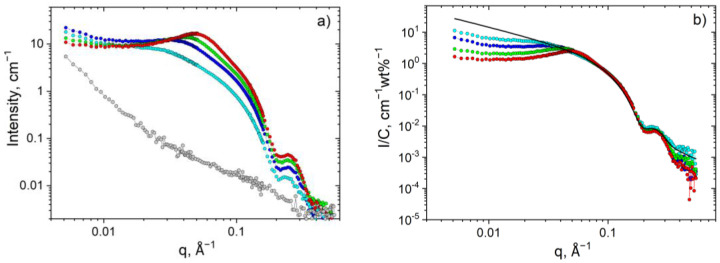
(**a**) SANS curves for HPG/potassium oleate/C_8_TAB systems in D_2_O containing 1 wt% HPG and different total surfactant concentrations C: 0 (**grey circles**), 1.6 (**cyan circles**), 3.3 (**blue circles**), 4.6 (**green circles**) and 6.6 wt% (**red circles**) at fixed molar ratio [potassium oleate]/[C_8_TAB] = 2.5. (**b**) SANS curves normalized by total surfactant concentration I/C vs. q. The solid line represents a fit by a form-factor of a cylinder with R = 19.2 Å.

The structure of the HPG/surfactant systems was studied by SANS. To enhance the contrast, D_2_O was used as a solvent (at pD = 11.0 ± 0.2 adjusted by addition of KOH). In this solvent, the surfactant micelles mainly contribute to the scattering when they are mixed with polymer chains [30], as shown in Figure 5a.

Figure 5 allows us to examine the evolution of the SANS curves with increasing surfactant concentration (at fixed polymer content). The higher-q part of the scattering curves can be well fitted by a form-factor of cylinder (solid line, Figure 5b) with a radius of 19.2 Å, which is close to the length of oleate tail (19 Å [6,58]). Therefore, HPG does not affect the local cylindrical structure of micellar aggregates.

**Figure 6:**
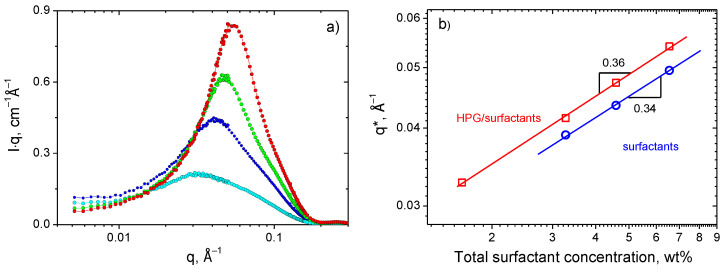
(**a**) The SANS curves represented by a Holtzer plot, I·q vs. q, for HPG/potassium oleate/C_8_TAB systems in D_2_O containing 1 wt% HPG and different total concentrations of the surfactants: 1.6 (**cyan circles**), 3.3 (**blue circles**), 4.6 (**green circles**) and 6.6 wt% (**red circles**) at fixed [potassium oleate]/[C_8_TAB] molar ratio equal to 2.5. (**b**) Position of the correlation peak, q*, as a function of surfactant concentration for the HPG/potassium oleate/C_8_TAB system containing 1 wt% HPG and for the corresponding potassium oleate/C_8_TAB system without polymer ([potassium oleate]/[C_8_TAB] molar ratio equal to 2.5).

The SANS curves of the HPG/surfactant system have a peak (Figure 5), which may be assigned to positional correlations arising from electrostatic repulsion between similarly charged micelles [59]. The position of the structure peak q* is more evident on a Holtzer or bending rod plot given by I(q)q ~ q dependence [60] (Figure 6a). It is seen that, with increasing surfactant concentration, the peak becomes more pronounced and shifts to higher q values indicating that the micelles come closer to each other.

Figure 6b shows the log-log dependence of the position of the correlation peak q* on the surfactant concentration. It is seen that q* scales as C_surf_^0.36^ in the presence of HPG and as C_surf_^0.34^ in its absence. These dependences are rather close to the power law expected for local hexagonal arrangement of the cylindrical micelles q* ~ C_surf_^0.5^ [61].

Note that in the presence of HPG the q* values are always higher than in pure surfactant solution (Figure 6). This indicates that polymer forces the WLMs to approach closer to each other. Similar behavior was previously observed upon addition of PVA to the same surfactant solution [30] and was explained by the microsegregation of polymer and micellar components resulting in the formation of polymer-rich and surfactant-rich domains. The expulsion of WLMs from polymer-rich regions leads to local increase in concentration of micelles in regions enriched by surfactants which is reflected in a decrease of the mean intermicellar distance (2π/q*) upon adding polymer to the solution. Similar reasons are valid for the present system as well.

The microphase separation can arise from a competition between (i) the tendency for segregation of polymer and surfactant components at a small scale resulting in the gain in energy of interactions and (ii) the long-range compatibilizing effect of micellar counterions that tend to displace in the whole volume of the system in order to gain in the translational entropy.

**Figure 7:**
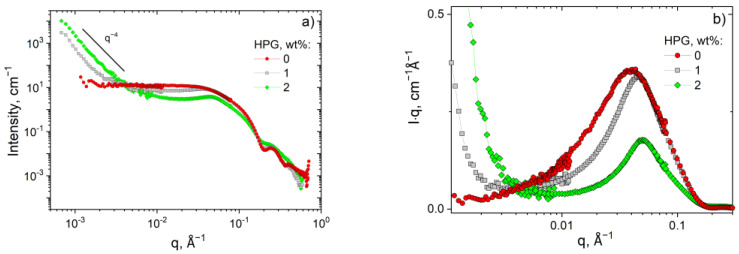
USANS curves in (**a**) I vs. q and (**b**) I·q vs. q (Holtzer plot) representations for HPG/potassium oleate/C_8_TAB systems in D_2_O containing 2.5 wt% potassium oleate, 0.8 wt% C_8_TAB and different concentrations of the HPG: 0 (**red circles**), 1 (**grey squares**), and 2 wt% (**green diamonds**).

Now let us consider the evolution of the SANS curves with increasing polymer concentration (at a constant number of surfactants). From Figure 7a we observe that the higher-q parts of the scattering curves coincide with each other confirming that the WLMs keep their local cylindrical structure intact despite the presence of polymer. At the same time, the added polymer shifts the peak position of the intermicellar correlation to higher q values (Figure 7b) meaning that the micelles approach each other (although the total concentration of surfactant does not change). As was discussed above, this is due to the expulsion of surfactants from the area occupied by polymer indicating the microphase separation. 

Figure 7a shows that the most pronounced effect of polymer is observed in the low-q region, where HPG induces a sharp increase in the scattering intensity that becomes more pronounced with increasing polymer concentration [62]. It represents one more indication of the formation of microphase separated structures. The q^4^ dependency in the low-q part of the scattering curve can be the Porod scattering from these structures. Similar behavior was observed for the aggregates of micelles of block-copolymers [63]. A strong upturn of the scattered intensity at low q (slope > 3) has been reported for various micro-heterogeneous systems as, for instance, polymer melts [64], percolated nanoemulsion colloidal gels [65], and bi-continuous nanoparticle gels obtained by solvent segregation [66,67].

Thus, the SANS/USANS data show that added polymer does not disturb the local cylindrical structure of WLMs, but induces a microphase separation with the formation of surfactant-rich and polymer-rich domains.

### 3.4. Rheological Properties

**Figure 8:**
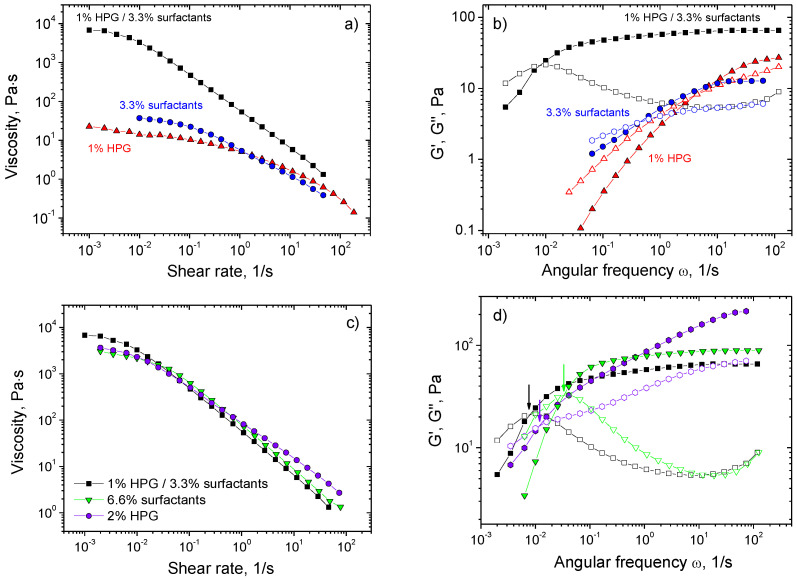
(**a**,**b**) Flow curves (**a**) and frequency dependencies of storage G′ and loss G″ moduli (**b**) for HPG/potassium oleate/C_8_TAB system (**black squares**) containing 1 wt% HPG, 2.5 wt% potassium oleate, 0.8 wt% C_8_TAB and its components: 1 wt% HPG (**orange triangles**) and surfactant solution with 2.5 wt% potassium oleate and 0.8 wt% C_8_TAB (**blue circles**) at 20 °C. (**c**,**d**) Flow curves (**c**) and frequency dependencies of storage G′ and loss G″ moduli (**d**) for the same HPG/potassium oleate/C_8_TAB system (**black squares**) containing 1 wt% HPG, 2.5 wt% potassium oleate, 0.8 wt% C_8_TAB in comparison with 2 wt% HPG (**violet circles**) and surfactant solution with 5 wt% potassium oleate and 1.6 wt% C_8_TAB (**green triangles**) at 20 °C. Arrows in (**d**) indicate G′, G″ crossover (G′ = G″).

Figure 8a,b presents the typical results of the measurements of rheological properties for the HPG/surfactant system and its components. Note that each of the components at the concentration under study forms a transient network as ascertained by the presence of a plateau on the frequency dependencies of the storage modulus G′(ω) (Figure 8b), which is characteristic of entangled solutions [14]. Therefore, the stress relaxation in all three systems is controlled by the chain reptation.

From Figure 8a,b one can see a clear synergistic effect of the mixing of polymer and surfactant solutions. Thus, the zero-shear viscosity η_0_ of the HPG/surfactant system is 6700 Pa.s, whereas η_0_ of polymer and surfactants alone is 22 and 37 Pa·s, respectively (Figure 8a). The plateau modulus equals 65 Pa for the mixed system and only 12 Pa for surfactants and 27 Pa for HPG solution taken separately (Figure 8b). The longest relaxation time, estimated as the inverse value of frequency at which G′ = G″, is 130 s for the mixed system and only 0.2 s and 2 s for polymer and surfactant components, respectively. Therefore, the most pronounced effect of mixing is produced on the viscosity and longest relaxation time: 180-fold and 65-fold increase in comparison with the components taken separately. This can be attributed to microphase separation leading to increasing local concentration of the components in each of the microphases, which produces more interpolymer and intermicellar entanglements. Moreover, in the case of WLMs, the local increase of surfactant concentration should induce the elongation of the micelles, since the length of micelles L augments with surfactant concentration C_surf_ as L ~ C_surf_^0.5^ [68]. Longer WLMs and larger number of entanglements within both polymer and surfactant microphases slow down the reptation thereby increasing both relaxation time and viscosity.

To reveal the impact of the local concentration of polymer and surfactant components, we compared the rheological properties of HPG/surfactants systems with those of its components (HPG and surfactants potassium oleate/C_8_TAB) taken separately at concentrations that are 2× higher than in the mixture. This concentration was chosen roughly assuming that the polymer and surfactant microphases occupy equal volumes (50% of the whole volume, each containing only polymer or surfactant). It was observed (Figure 8c,d) that the rheological characteristics of 2× concentrated polymer and surfactant solutions become close to the mixed HPG/surfactant system. This result counts in favor of our hypothesis that this is the concentrating of each of the components of HPG/surfactant system in the corresponding microphases, which is mainly responsible for the enhancement of the rheological properties. Note that the zero-shear viscosity and the longest relaxation time of the 2× concentrated polymer and surfactant solutions are still somewhat lower than those of mixture in contrast to the plateau modulus, which is somewhat higher. These data are also consistent with our hypothesis, since the microphase separation restricts the number of entanglements between different components (polymer and micellar chains) which is reflected in somewhat smaller plateau modulus. Note that our assumption of pure polymer and surfactant microphases overestimates the extent of segregation.

Thus, simple mixing of WLMs and polymer solutions induces a large synergistic enhancement of the viscosity and relaxation time, which can be attributed to the increase in local concentration of polymer and surfactants within the microphase separated domains.

## 4. Conclusions

For the first time, binary transient networks composed of a network of entangled long wormlike surfactant micelles and a network of entangled polysaccharide chains were prepared. The WLMs forming the micellar network were composed of a mixture of anionic (potassium oleate) and cationic (C_8_TAB) surfactants with a large excess of anionic surfactant, that is they were highly negatively charged. The polymer network was formed by very long chains of uncharged industrially important polysaccharide HPG. By ^1^H NMR spectroscopy it was shown that there are no attractive interactions between the polymer and surfactant components, since the chemical shifts of the peaks in the spectra of the dual networks do not differ from those of its individual components taken separately. The dual network system demonstrates a good compatibility in a wide range of polymer and surfactant concentrations. This may be due to highly charged WLMs which prevent macrophase separation, because it will decrease the translational entropy of their counterions when they will be confined in a small volume of one of the phases. At the same time, by SANS combined with USANS, a microphase separation was detected. It is manifested in a sharp upturn of the scattering curves at ultra-small angles (q < 0.003 Å^−1^) and in the shift of the peak arising from intermicellar correlations at ca. 0.04–0.05 Å^−1^ to smaller q values upon addition of polymer. The upturn suggests the appearance of large inhomogeneities, whereas the shift of the peak indicates the decrease of the mean distances between the WLMs, when polymer is added. Meanwhile, the polymer does not affect the local cylindrical structure of the WLMs. These data suggest the expulsion of the surfactant from the area occupied by polymer and its concentrating in the surfactant-rich microdomains.

One of the most important findings of this study is the observation of the synergistic enhancement of the rheological properties upon mixing of polymer and WLM solutions resulting in a 180-fold increase of viscosity and a 65-fold increase of the longest relaxation time in comparison with the individual components. This behavior was attributed to local concentration of polymer and surfactants within polymer-rich and surfactant-rich microphases inducing the elongation of WLMs and the increase of intermicellar and interpolymer entanglements. A synergistic enhancement of rheological characteristics permits to reduce significantly the amount of polymer and surfactants required to achieve the desired rheological properties, which is profitable from both economical and ecological points of view. Taking into account the wide industrial application interest of the polymer under study, these findings pave a promising way for developing various new commercial formulations, for instance, new fracturing fluids for oil recovery or new personal care products with improved properties.

## Figures and Tables

**Table 1:** Table of integrals in ^1^H NMR spectra of HPG/potassium oleate/C_8_TAB system and its components: HPG and potassium oleate/C_8_TAB mixture *.

Peak **	Position, ppm	Integral		
		HPG/Potassium Oleate/C_8_TAB	HPG	Potassium Oleate/C_8_TAB
18, 8′	0.85–0.89	7.06		7.01
HPG hydroxypropyl	1.15	3.32	2.92	
3′–7′, 4–7, 12–17	1.26–1.29	42.09		42.21
3	1.52	3.48		3.57
2′	1.72	1.32		1.39
8, 11	2.00	6.65		6.68
2	2.10	3.42		3.44
N-CH_3_	3.10	6.13		6.17
1′	3.25	1.38		1.37
HPG CH_2_ CH	3.45–4.11	10.72	10.73	
HPG m1	4.73	0.60	0.60	
HPG g1	5.01	0.40	0.39	
HPG g1	5.18	0.09	0.07	
9, 10	5.32	3.31		3.31

* Concentrations: 0.7 wt% HPG, 1.25 wt% potassium oleate, 0.395 wt% C_8_TAB (molar ratio [potassium oleate]/[C_8_TAB] = 2.5). Solvent: D_2_O. Temperature: 70 °C. ** Peak assignments are indicated in Figure 4.

## Data Availability

The data presented in this study are openly available. ILL data are curated under DOI: 10.5291/ILL-DATA.9-11-2009.
